# Characterizing hypertension in activated PI3K delta syndrome: Findings from Epic Cosmos

**DOI:** 10.70962/jhi.20250263

**Published:** 2026-10-26

**Authors:** Brianna Santiago, Jennifer Keelin, Xiaoqi Sun, Christopher Chang

**Affiliations:** 1 Herbert Wertheim College of Medicine, Florida International University, Miami, FL, USA; 2 Office of Human Research, Memorial Healthcare System, Hollywood, FL, USA; 3 Division of Immunology, Allergy and Rheumatology, Joe DiMaggio Children’s Hospital, Hollywood, FL, USA

## Abstract

APDS is a rare inborn error of immunity with comorbidity patterns that have yet to be characterized at a large scale. Using Epic Cosmos, a de-identified electronic health record database, we conducted a retrospective, cross-sectional analysis of 866 patients identified by structured diagnosis codes (ICD-10: D84.81; SNOMED: 793412000). Findings revealed that hypertension was coded as affecting 28.5% of the cohort, with age-stratified analyses demonstrating higher prevalence among younger APDS patients compared with age-matched non-APDS peers. Among patients aged ≤50 years, hypertension prevalence was 24.05% in the APDS group compared with 10.89% in the age-matched non-APDS population (prevalence ratio 2.21, 95% CI, 1.90–2.57, P < 0.0001). This association remained prominent in a more specific APDS sensitivity cohort in which the base population required an APDS diagnosis, alongside an allergy and immunology encounter. The findings of this paper provide large-scale characterization of APDS and identify hypertension, particularly among younger APDS patients, as a potentially underrecognized comorbidity that warrants further investigation.

## Introduction

Although often discussed in the context of immunodeficiency, APDS is more accurately characterized as a dysregulation of the PI3K pathway that plays a critical role in the normal function of B and T cells. Activated PI3K delta syndrome (APDS) is newly recognized as an inborn error of immunity resulting in a wide range of clinical manifestations (1). Researchers have described the PI3K pathway as relying on the “Goldilocks effect,” in which insufficient and excessive activation results in greater susceptibility to recurrent respiratory infections, lymphoproliferation, autoimmunity, and malignancy (2).

This syndrome is caused by germline mutations in either PIK3CD, leading to a gain-of-function in the p110δ catalytic subunit (APDS1), or in PIK3R1, resulting in loss-of-function of the p85α regulatory subunit (APDS2). In the PI3K pathway, p110δ catalyzes the conversion of PIP2 to PIP3, while p85α acts as a regulatory subunit that restrains p110δ activity. Therefore, gain-of-function variants in PIK3CD or loss-of-function variants in PIK3R1 result in excessive PIP3 production and constitutive activation of downstream AKT/mTOR signaling. B and T cell function are impacted via defective class-switching, impaired T cell apoptosis, and poor B cell memory (2).

Diagnosis of APDS is established by identifying pathogenic variants in PIK3CD or PIK3R1 (3), most commonly through primary immunodeficiency, immune dysregulation, or oncologic predisposition gene panels. Clinical features that may prompt genetic evaluation include recurrent sinopulmonary infections, lymphoproliferation, cytopenias, bronchiectasis, splenomegaly, and abnormal immunoglobulin profiles (4, 5).

Emerging evidence suggests that APDS may encompass a broader clinical burden than previously recognized. In addition to recurrent infections and lymphoproliferation, comorbid conditions such as gastrointestinal manifestations, autoimmunity, and malignancy have been reported in previous cohorts; however, hypertension has not been systematically described (6). Moreover, with the recent FDA approval of leniolisib and the growing use of targeted therapies, there is a need to better understand treatment patterns and their impact within the APDS population.

While prior APDS registry studies have provided rigorous and detailed characterization of molecularly confirmed APDS cohorts, large-scale real-world EHR-based studies may offer complementary insight into comorbidities and treatment documentation across broader healthcare settings (5, 6). This study leverages Epic Cosmos, an EHR dataset, to provide the most comprehensive characterization of APDS to date, highlighting demographic trends, treatment utilization, and novel associations such as an increased prevalence of hypertension.

## Results

**Figure 1.**
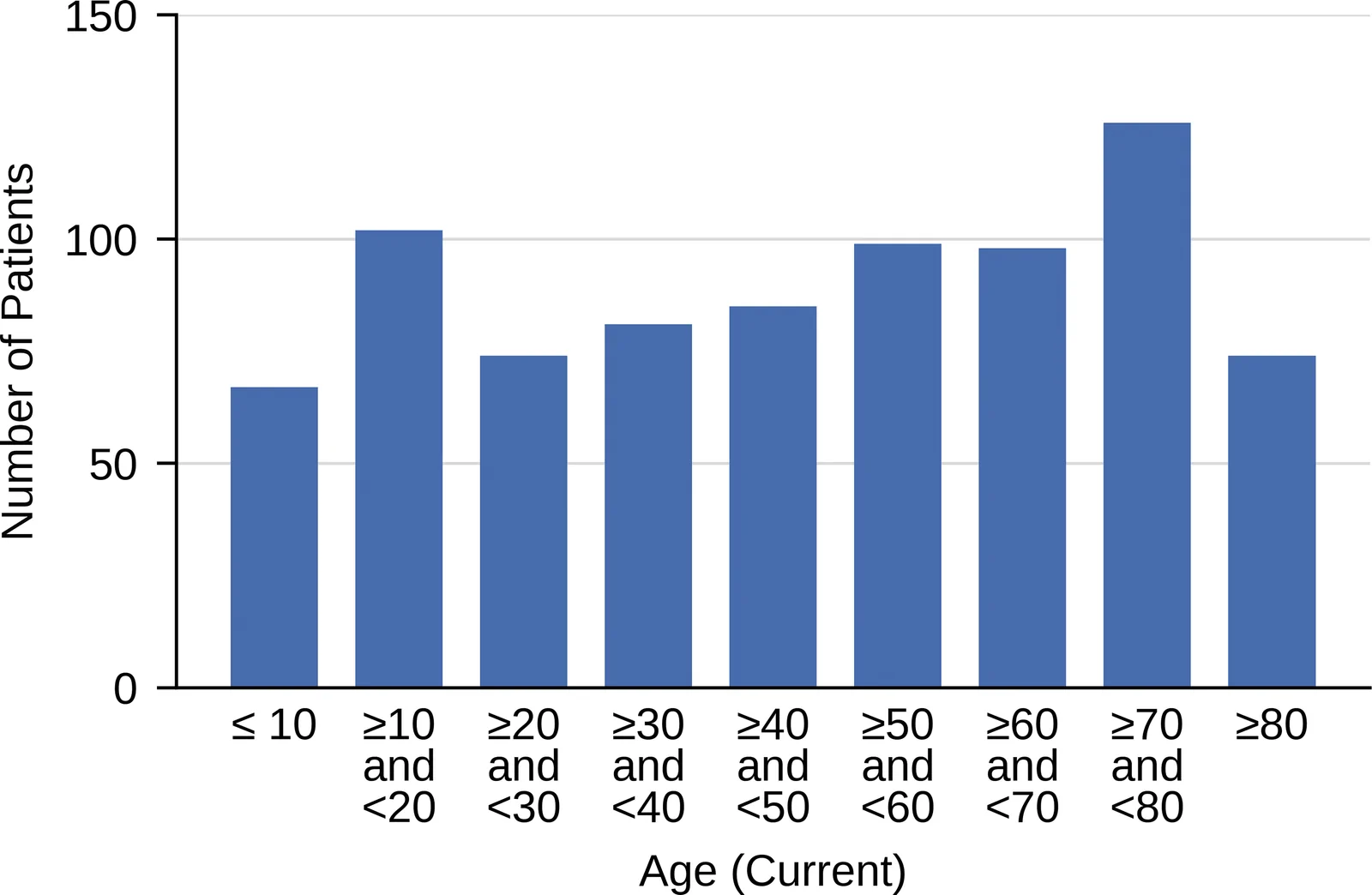
Age distribution of APDS patients in the Epic Cosmos cohort.

A total of 866 patients with an ICD-10 diagnosis of APDS were identified within the Epic Cosmos EHR database. A slight majority of the cohort (55.7%) were female. The racial composition was predominantly white (73.4%), with smaller representations of African Americans/Black (10.1%), Asian (3.1%), and other racial groups (9.76%). Because APDS is often recognized during childhood or adolescence but is increasingly identified across the lifespan, we examined the current age distribution of APDS-coded patients in the Epic Cosmos cohort. The age distribution is reflective of prevalent cases within the EMR system rather than age at diagnosis or disease onset (Fig. 1).

**Figure 2.**
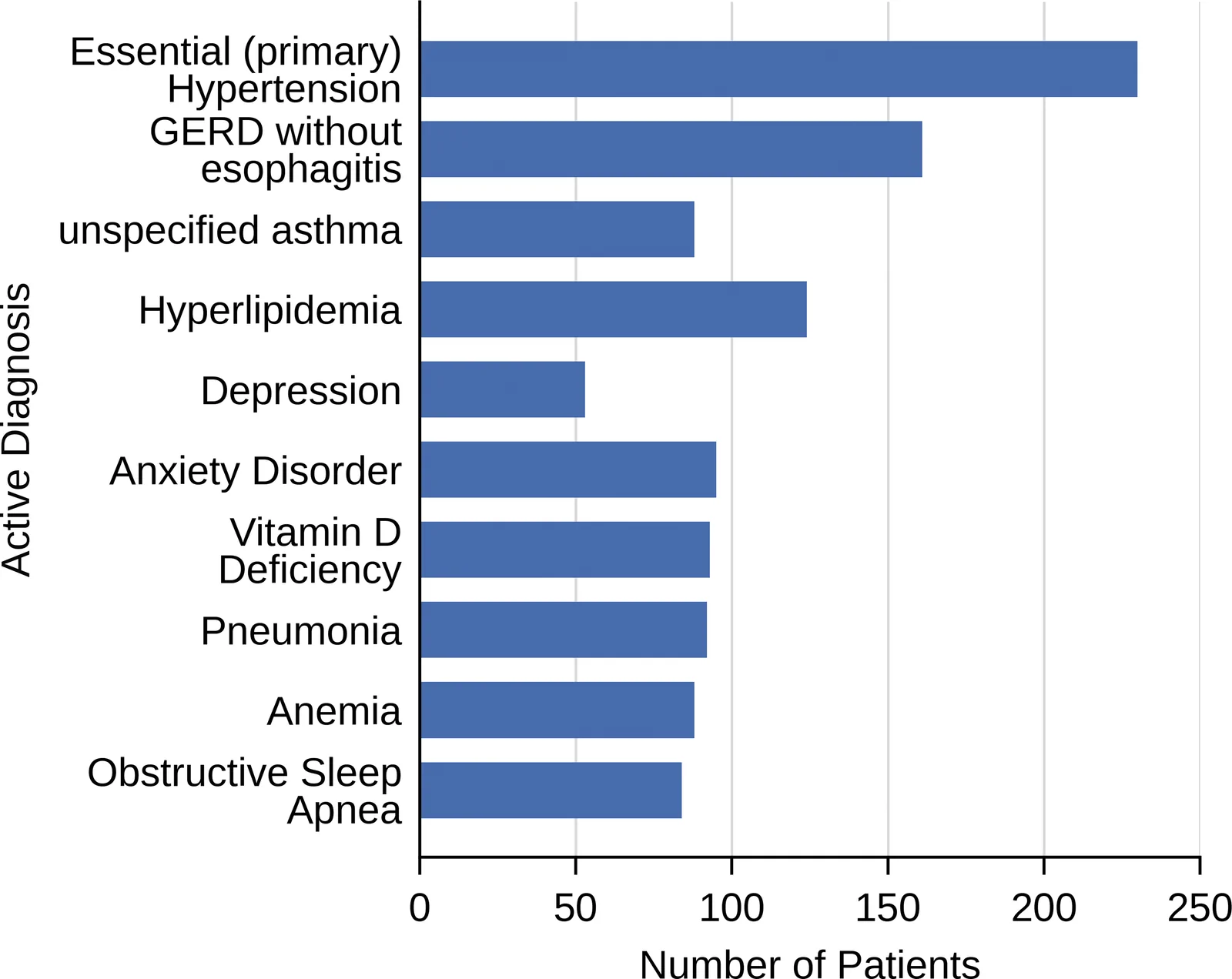
Top 10 reported active diagnoses among APDS patients.

A review of active diagnoses showed a wide range of comorbidities often seen in people with immune system issues or chronic health problems. The most common condition was hypertension, affecting 28.5% of patients. This finding is further supported by a sensitivity analysis restricted to patients with encounters in allergy and immunology departments, which yielded 152 patients with a similar hypertension prevalence (34 patients, 22.37%) (Fig. 2).

Age-stratified analyses demonstrated that hypertension occurs earlier and with higher prevalence in APDS patients compared with non-APDS peers (Table 1). The greatest relative differences were observed among adolescents and young adults, whereas hypertension prevalence increased steadily with age in both populations. Specifically, the proportion of APDS patients with hypertension diverged in early adulthood (20–30 years, 20.3% vs. 7.3% in non-APDS patients) and remained consistently higher across age groups. By middle adulthood (40–50 years), 40.9% of APDS patients were hypertensive compared with 27.1% of non-APDS peers.

**Table 1.** Age at encounter-stratified prevalence of hypertension in APDS patients compared with non-APDS Epic Cosmos peers using chi square

Age group	% HTN in APDS cohort	% HTN in non-APDS Epic Cosmos peers	P value
10–19 (*n* = 131)	12.9	2.06	<0.0001
20–29 (*n* = 118)	20.3	7.3	<0.0001
30–39 (*n* = 129)	29.5	15.0	<0.0001
40–49 (*n* = 132)	40.91	27.1	0.00037
50–59 (*n* = 141)	63.8	41.1	<0.0001
60–69 (*n* = 168)	75.8	53.8	<0.0001
70–79 (*n* = 181)	80.1	65.6	<0.0001
80–89 (*n* = 84)	85.7	70.0	0.0017

In considering the lack of genomic testing available on Epic Cosmos, we assessed the prevalence of at least one APDS clinical manifestation within the 866-patient cohort, with 750 patients (86.3%) having at least one APDS manifestation and 262 patients (30.1%) having two or more APDS clinical manifestations. Lymphadenopathy was present in 571 patients (65.7%), cytopenia in 431 (39.2%), splenomegaly in 146 (16.8%), bronchiectasis in 73 (8.4%), and pneumonia in 236 (18.7%). These findings are consistent with previously reported prevalence rates of APDS and support clinical utility of this dataset, capturing a true APDS population (5, 6). Additional common diagnosis coded in Epic Cosmos for the APDS-coded patients were GERD in 19.9% and hyperlipidemia in 15.4%. The observed combination of chronic inflammation and respiratory infections supports previously established immune challenges in APDS, relating to both the disease itself and the treatments being used.

Age-stratified prevalence estimates are presented descriptively because the exported aggregate output did not include the full underlying numerator and denominator data needed to calculate confidence intervals for prevalence differences between groups. In addition, some subgroup outputs may be limited by Epic Cosmos privacy thresholds for small patient counts.

To further investigate the findings of prominent differences in prevalence of hypertension in younger APDS populations, an additional analysis was performed to assess the difference in hypertension prevalence when the age groups are restricted to individuals aged ≤50 years (Table 2). Among APDS patients aged ≤50 years, 127 of 528 (24.05%; 95% CI, 20.60–27.88%) had hypertension compared with 17,543,972 of 161,143,655 (10.89%; 95% CI, 10.88–10.89%) individuals in the age-matched non-APDS Epic Cosmos population, corresponding to a prevalence ratio (PR) of 2.21 (95% CI, 1.90–2.57; P < 0.0001). The association persisted in the more restrictive Allergy and Immunology sensitivity cohort, in which 30 of 144 patients aged ≤50 years had hypertension (20.83%; 95% CI, 14.50–27.60%), corresponding to a PR of 1.91 compared with the age-matched non-APDS population (95% CI, 1.39–2.63; P < 0.0001).

**Table 2.** Comparison of hypertension APDS patient cohort, sensitivity cohort, and age-matched non-APDS population below ≤50 years

Population, age ≤50	HTN, *n*	No HTN, *n*	Total, *N*	HTN prevalence (95% CI)	Prevalence ratio (95% CI)	P value
APDS	127	401	528	24.05% (20.60–27.88%)	**2.21** (1.90–2.57)	<0.0001
APDS sensitivity cohort	30	114	144	20.83% (14.50–27.60%)	**1.91** (1.39–2.63)	<0.0001
Non-APDS Epic Cosmos population	17,542,972	143,599,683	161,143,655	10.89% (10.88–10.89%)	-	-

The bold values in Table 2 highlight the prevalence ratios comparing hypertension prevalence in the APDS cohorts with the age-matched non-APDS population, representing the primary comparative findings of this analysis.

Therapeutic data captured in the EHR revealed variable prescription patterns in pharmaceutical management among APDS patients. The most frequently documented disease-directed therapies included intravenous immunoglobulin (IVIG) in 147 patients and leniolisib, a PI3Kδ inhibitor recently approved for APDS, in 107 patients. Sirolimus, an mTOR inhibitor, was prescribed in 73 cases, whereas calcineurin inhibitors such as tacrolimus were reported in 39 patients.

Mycophenolate mofetil use was less common (*n* = 28), and all other targeted PI3K inhibitors (e.g., alpelisib, idelalisib) were observed in <10 patients each. Notably, most patients in the cohort (*n* = 762) had no record of receiving any of these disease-directed agents within Epic Cosmos. In addition, subcutaneous immunoglobulin (SCIG) counts were below the reporting threshold (<10), which may reflect how SCIG is predominantly self-administered at home and not captured in hospital-based EHR data. Treatment utilization in this cohort should be interpreted as a reflection of what is recorded within the Epic Cosmos network, rather than a comprehensive account of all disease-directed therapies these patients are receiving.

**Figure 3.**
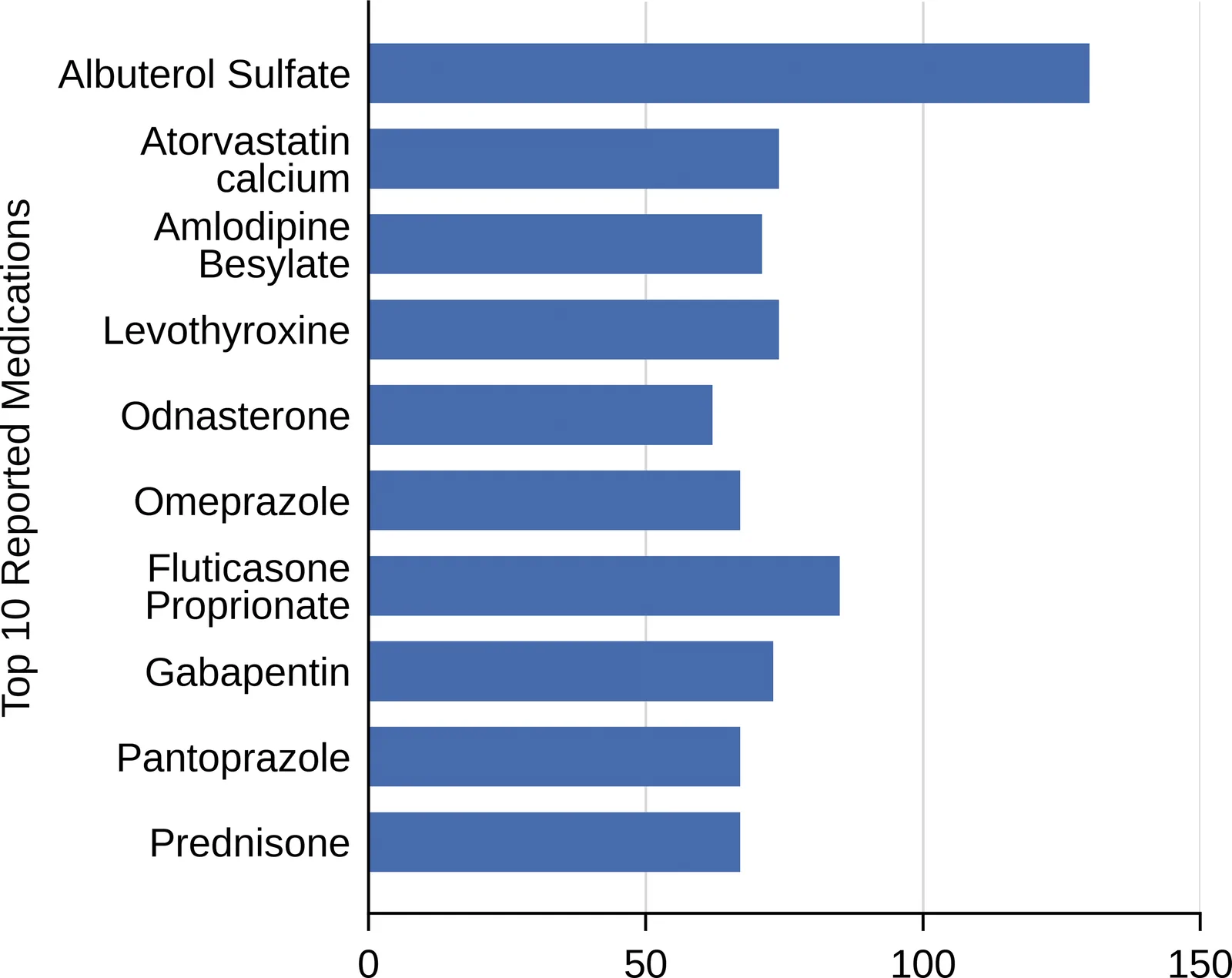
Top 10 medications prescribed overall in the APDS patient cohort.

When evaluating the top reported medications overall, therapies most often reflect management of comorbid conditions such as hypertension and hyperlipidemia, rather than APDS-specific interventions (Fig. 3). The most frequently prescribed drugs included albuterol sulfate (*n* = 130), atorvastatin (*n* = 74), amlodipine (*n* = 71), levothyroxine (*n* = 74), fluticasone propionate (*n* = 85), and prednisone (*n* = 67). To further contextualize the high prevalence of hypertension identified in the APDS cohort, documentation of antihypertensive medications was evaluated. Overall, 311 (35.8%) patients within the cohort were being treated with antihypertensives (Table 3). It is important to note that the proportion of patients with documented prescriptions for antihypertensive medications exceeded the proportion of those with a hypertension diagnosis (28.5%), which may imply that the medications are prescribed for indications other than hypertension and, while informative, may not be an accurate surrogate measure of hypertension prevalence.

**Table 3.** Prevalence of antihypertensive prescriptions in the overall APDS Cohort

Antihypertensive	Count of APDS patients	Percent of cohort
Amlodipine	184	21.2
Losartan	130	14.9
Lisinopril	142	16.3
Metoprolol	215	24.7
Hydrochlorothiazide	132	15.2
Spironolactone	80	9.21
Prescription of any antihypertensive	311	**35.8**
None of the above	456	52.5

In Table 3, the bold value (35.8%) highlights the overall proportion of APDS patients with a documented prescription for at least one antihypertensive medication, distinguishing this aggregate measure from the individual medication categories.

**Figure 4.**
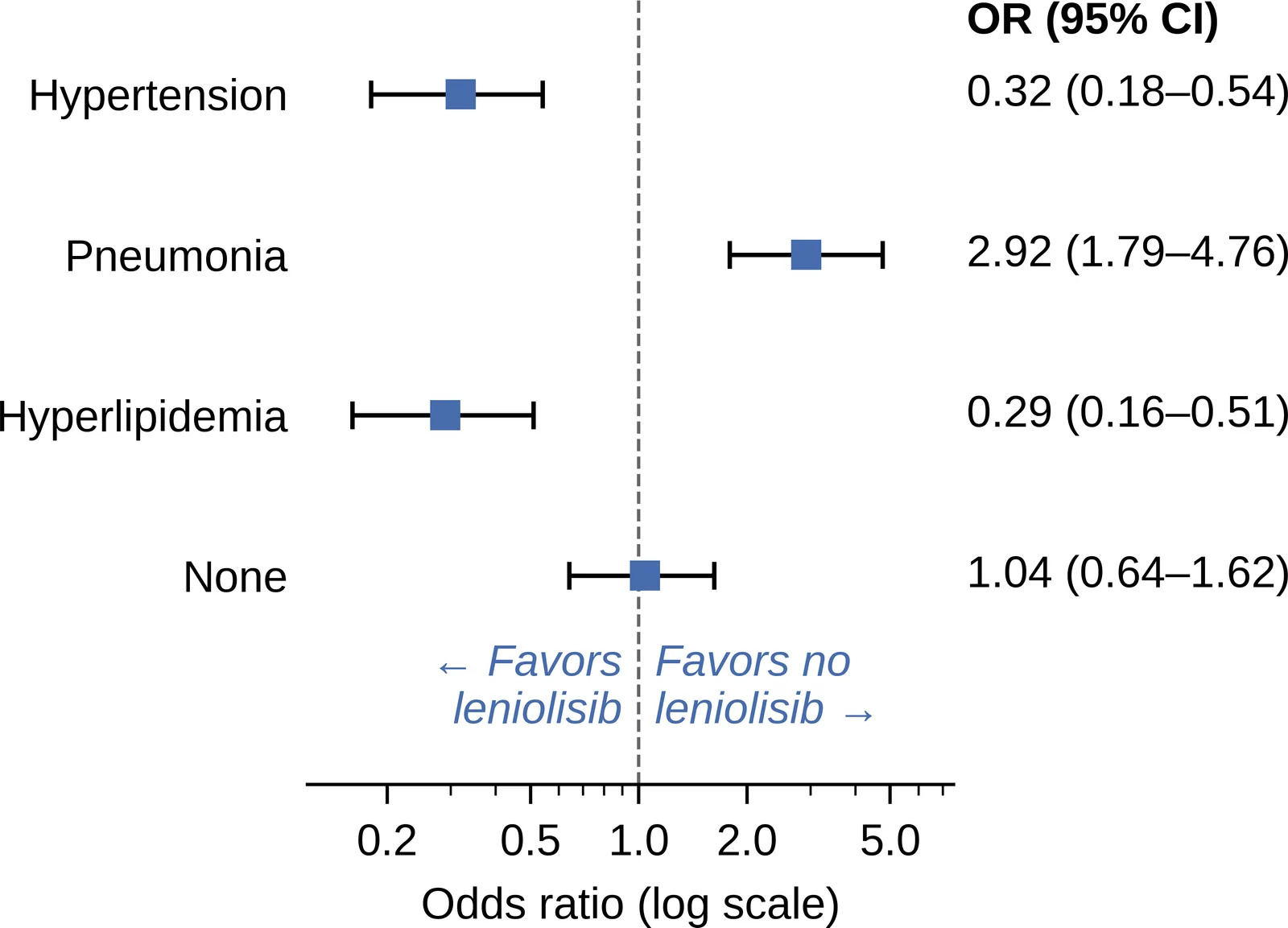
Comparing comorbidities in leniolisib vs. non-leniolisib APDS patients.

To further characterize APDS patients with documented disease-directed therapy, comorbidities such as hypertension, pneumonia, and hyperlipidemia were compared between patients with and without documented leniolisib treatment (Fig. 4). Odds of hypertension were significantly lower in the leniolisib group (OR 0.32, 95% CI, 0.18–0.54, P < 0.001), as were odds of hyperlipidemia (OR 0.29, 95% CI, 0.16–0.51, P < 0.001). In contrast, odds of pneumonia were significantly higher among leniolisib-treated patients (OR 2.92, 95% CI, 1.79–4.76, P < 0.001).

## Discussion

In this study, Epic Cosmos has been leveraged to explore the demographic and clinical characteristics of a historically underrecognized immunodeficiency by capturing the largest, multi-institutional cohort of individuals with APDS. The ability to gather such an extensive dataset is valuable to demonstrating the power of Epic Cosmos in investigating rare diseases (Figs. 1 and 2).

Most notably, hypertension was the highest reported comorbidity among APDS patients in the cohort, suggesting cardiovascular comorbidity may represent an underrecognized aspect of the disease phenotype that is yet to be discussed in previous literature. While hypertension is common in the general US adult population, there is a greater prevalence of hypertension within the younger groups of the Epic Cosmos APDS cohort despite many inborn errors of immunity being recognized earlier in life.

Within Epic Cosmos, hypertension is captured as an active diagnosis code, which reflects clinician-assigned chronic diagnosis rather than a single elevated blood pressure reading. With that distinction in mind, age-stratified analyses demonstrated that hypertension occurs with higher prevalence in APDS patients compared with non-APDS peers across all age groups. Yet, statistical significance was primarily demonstrated in APDS coded patients below the age of 50.

Notably, the greatest relative differences were observed among adolescents and young adults, indicating that the higher prevalence of hypertension in the APDS cohort is not simply attributable to an older patient population. Rather, the finding raises the possibility of an earlier cardiovascular burden in APDS coded patients. This interpretation was supported by the analysis restricted to patients aged ≤50 years, in which hypertension prevalence remained more than twofold higher among APDS patients compared with the age-matched non-APDS population (24.05% vs. 10.89%; PR 2.21, 95% CI, 1.90–2.57; P < 0.0001). The association persisted in the more restrictive Allergy and Immunology sensitivity cohort, in which hypertension prevalence was 20.83%, corresponding to a 1.91-fold higher prevalence than the non-APDS population (95% CI, 1.39–2.63; P < 0.0001). Although the magnitude of the association was attenuated in the sensitivity cohort, its persistence after restricting both age and specialty encounter criteria strengthens the finding that the elevated hypertension prevalence observed in APDS may not solely be explained by the inclusion of older patients or by the broader APDS case definition.

Whereas hypertension has not previously been widely described as part of the APDS phenotype, emerging literature suggests a broader systemic impact of PI3Kδ hyperactivation beyond immune dysfunction. Experimental data in a hypertensive rat model support the observation that PI3K pathway modulation influences blood pressure regulation as it was demonstrated that inhibiting the PI3K/Akt pathway reduced oxidative stress, improved endothelial function, and significantly lowered blood pressure (7). Similar mechanisms have been described in preeclampsia, where PI3K/Akt dysregulation contributes to vascular dysfunction and hypertension (8). Renal disease and autoimmune complications may contribute to secondary hypertension in some APDS patients and should be evaluated in future studies with greater clinical granularity. The large-scale Epic Cosmos analysis extends these isolated clinical observations to a population level, demonstrating that hypertension occurs with higher prevalence in APDS coded patients compared with non-APDS peers.

Prior literature, along with our findings, suggests that pharmacologic PI3Kδ inhibition may restore vascular homeostasis by mitigating the downstream endothelial and oxidative stress effects of pathway overactivation. With identification of hypertension as a potential prominent comorbidity in APDS, we explored whether documented disease-directed therapy was associated with any difference in hypertension prevalence. Leniolisib-treated patients had a markedly lower prevalence of hypertension (23.1% vs. 48.8%), with an odds ratio of 0.315 (P = 0.0003). This association should be interpreted cautiously because treatment timing, disease severity, age, and prescribing patterns could not be fully assessed in the current dataset. In addition, selection bias in prescribing cannot be excluded. Taken together, these results highlight both an underrecognized burden of hypertension in APDS and a hypothesis-generating association between leniolisib documentation and lower hypertension prevalence that warrants prospective validation. Leniolisib, FDA-approved in 2023, was the most frequently documented targeted therapy in the APDS cohort, recorded in 107 patients (12.3%). This reflects integration of target therapies into clinical practice and highlights a strength of Epic Cosmos in capturing emerging treatment patterns. The lower prevalence of hypertension and hyperlipidemia in the leniolisib-treated group may also reflect prescribing patterns, age distribution, or other unmeasured confounders rather than a direct treatment effect. The differences in pneumonia prevalence may be influenced by treatment selection bias, where patients with more severe infectious histories may have been good candidates for targeted therapy. Although these findings do not establish a causal relationship, they raise the possibility that PI3K pathway inhibition may influence the early cardiovascular phenotype observed in APDS. Together, these findings position hypertension as a potential comorbidity that warrants prospective evaluation in APDS, particularly in studies with longitudinal treatment data, blood pressure measurements, and adjustment for disease severity and medication exposure.

It is important to acknowledge several treatment-related confounders when interpreting the association with hypertension. For example, sirolimus, prescribed in 73 patients, is well documented to cause or worsen hypertension. Therefore, patients previously treated with sirolimus may carry a diagnosis of hypertension that cannot be exclusively attributed to the APDS diagnosis (9). Corticosteroids and mycophenolate mofetil, used for the autoimmune manifestations of APDS, are also associated with secondary hypertension. Lastly, hematopoietic stem cell transplantation (HSCT), frequently used for severe APDS, is a well-established risk factor for hypertension. Whereas the contribution of HSCT to hypertension in APDS patients could not be assessed from the current dataset, it does warrant investigation in future research. Other potential contributors include renal disease, autoimmune disease such as lupus nephritis, lymphoma and oncologic therapy, and underlying disease severity. Because Epic Cosmos does not provide sufficient granularity regarding medication timing, cumulative exposure, transplant history, renal involvement, lymphoma treatment, or temporal onset of hypertension, these factors could not be fully adjusted for in the current analysis.

The high prevalence of hypertension in young APDS coded patients highlights the need for multidisciplinary management that extends beyond specialty-specific care involving immunologists. Recognition of the cardiovascular manifestation of APDS by primary care physicians and clinicians can warrant routine blood pressure monitoring at younger ages for this population and possible treatment. Future prospective studies should evaluate whether PI3Kδ inhibition influences the early cardiovascular phenotype observed in APDS and whether targeted therapy modifies long-term hypertension risk. These findings support development of a model in which immunologists, primary care clinicians, and cardiologists can work together to provide quality, comprehensive care for patients with APDS.

### Limitations

This study has several limitations inherent to EHR-based research. First, cohort identification relied on structured diagnosis codes, and genetic confirmation of APDS through PIK3CD or PIK3R1 testing was not available in Epic Cosmos. Although the Allergy and Immunology sensitivity cohort and the presence of APDS-associated manifestations support the plausibility of the cohort, these approaches cannot substitute for molecular confirmation. Second, the dataset is cross-sectional and does not establish temporal relationships among APDS diagnosis, medication exposure, hypertension onset, and treatment initiation. Third, medication data reflect therapies documented within participating health systems and may not capture medications administered externally, at home, or outside the Epic Cosmos network. This is particularly relevant for subcutaneous immunoglobulin (SCIG), which is frequently administered at home and was below the Epic Cosmos reporting threshold in this cohort. The low values of SCIG may suggest that medication frequencies reported in Epic Cosmos and, subsequently, this analysis require further review because of incomplete medication capture in the EHR. Fourth, the dataset lacks clinical granularity regarding genotype, disease severity, blood pressure measurements, renal disease, lymphoma treatment, HSCT history, and cumulative medication exposure. Finally, patient count suppression and missing demographic data may limit interpretation of subgroup analyses.

Because Epic Cosmos provides aggregate-level outputs rather than patient-level data, multivariable analysis adjusting for age, medication exposure, renal disease, HSCT history, lymphoma, and other potential confounders could not be performed. Consequently, the reported odds ratios represent unadjusted associations. These findings should be interpreted as hypothesis-generating and serve as a foundation for future patien- level investigations.

### Conclusion

This study leverages the largest national EHR dataset to date via Epic Cosmos to characterize APDS; identifying hypertension as the most frequently documented comorbidity and highlighting an underrecognized aspect of the APDS phenotype This finding is notable given that APDS has traditionally been recognized through infectious, lymphoproliferative, and immune dysregulatory manifestations, whereas cardiovascular comorbidity has been less well characterized.

Our age-stratified analyses demonstrate that the hypertension diagnosis is at higher prevalence across all APDS age groups when compared with non-APDS Epic Cosmos peers, which brings forth a potentially underrecognized cardiovascular burden or manifestation in this population. Exploratory analyses demonstrated an association between documented leniolisib treatment and lower odds of hypertension. These findings should be interpreted as hypothesis-generating given the potential for treatment selection bias and unmeasured confounding variables.

Ultimately, Epic Cosmos usage allowed us to investigate associations on a large scale for a rare condition such as APDS. Moreover, this study helps establish a foundation for additional hypothesis-driven studies regarding cardiovascular risk, treatment effectiveness, and health disparities in APDS patients.

## Materials and methods

This retrospective, cross-sectional study utilized de-identified patient data from Epic Cosmos, a multi-institutional electronic health record (EHR) database that aggregates real-world clinical data from participating health systems across the USA. Data from 7/11/2020 to 9/30/2025 were extracted in June 2026, from all contributing health systems. Patients were identified using ICD-10 code D84.81 and the corresponding SNOMED code 793412000 for APDS. To be included, patients were required to have at least one clinical encounter and complete demographic information. Epic Cosmos software omits patients from category specific totals if a demographic field is missing or suppressed to protect patient privacy. Epic Cosmos uses patient-level linkage and deduplication processes to consolidate records for the same individual across participating health systems. Accordingly, the analytic base population consisted of distinct patients rather than encounters or admissions, reducing the potential for multiple counting of individuals receiving care across different institutions. Because Epic Cosmos provides aggregate-level data for analysis, patient-level multivariable regression adjusting for demographic characteristics, comorbidities, and medication exposures was not feasible. Lastly, Epic Cosmos is a dynamic HER database in which historical patient counts may change as contributing health systems update their records.

The following demographic and clinical variables were collected: age, legal sex, race, insurance status, US census region, and active diagnoses. Descriptive statistics were used to characterize the APDS patient population, comorbidities, and treatment documentation. For comparisons between leniolisib-treated and non-leniolisib APDS patients, odds ratios with 95% confidence intervals were calculated, and significance was assessed using z-scores. Age at encounter-stratified hypertension prevalence between APDS patients and non-APDS Epic Cosmos peers was compared using χ-square testing, with prevalence estimates and P values reported for each age group.

To further evaluate if the observed association was driven by the age distribution of the overall APDS cohort, an additional analysis was performed among patients aged ≤50 years. Prevalence of hypertension in the overall APDS cohort and the APDS sensitivity cohort restricted to patients with Allergy and Immunology encounters was compared with the age matched non-APDS Epic Cosmos population. Prevalence ratios with 95% confidence intervals were calculated, along with an assessment of statistical significance using chi square testing in Epic Cosmos.

## Data Availability

Data were derived from Epic Cosmos, a secure de-identified electronic health record database. Aggregated data and analysis outputs are available within the manuscript. Epic Cosmos data are governed by institutional agreements and cannot be publicly shared. Access to the dataset can be granted through Epic upon request.
